# Characterisation of a New Bioactive Glass-Containing Fluoride Varnish

**DOI:** 10.3390/ma19091766

**Published:** 2026-04-26

**Authors:** Emily Thambi, Saroash Shahid, Melissa Tiskaya, Karin A. Hing, Swati Nehete, Robert Hill

**Affiliations:** 1 Institute of Dentistry, Faculty of Medicine and Dentistry, Queen Mary University of London, Mile End Road, London E1 4NS, UK; 2 School of Engineering and Materials, Queen Mary University of London, London, E1 4NS, UK

**Keywords:** bioactive glass, dental varnish, bioactive glass varnish, tooth remineralisation, calcium, phosphate, fluoride, fluorapatite, prevention of caries

## Abstract

**Objectives**: This study characterised the bioactive properties (i.e., ion release, pH rise, and apatite formation) of a newly developed Voco Profluorid + BioMin F varnish. Three additional varnishes were investigated for comparison: Clinpro™ White Varnish (3M™, St. Paul, MN, USA), MI Varnish (GC, Tokyo, Japan), and Profluorid varnish (VOCO GmbH, Cuxhaven, Germany). The Clinpro™ White and MI varnishes were chosen for comparison due to their similar composition of active ingredients. Profluorid served as a standard fluoride-only varnish reference. **Methods**: Dental varnish ingredients were characterised using ATR-FTIR, XRD, and ^19^F and ^31^P MAS-NMR. Coated coverslips were immersed in Tris buffer and artificial saliva (pH 4.0 and 7.0) for 2–24 h. Ion release was analysed using ICP-OES and a fluoride ion-selective electrode whilst monitoring pH changes. Post-immersion, coverslips were analysed by XRD and MAS-NMR to assess possible apatite formation. **Results**: XRD and ^19^F MAS-NMR detected NaF in all four varnishes. BioMin F varnish showed a ^31^P peak matching BioMin F glass, with an additional brushite peak, indicating partial reaction of the bioactive glass (BAG) with rosin resin water. All varnishes released fluoride and calcium, but only BioMin F and MI varnishes released phosphate, which is essential for the formation of calcium fluorapatite. Post-immersion analysis confirmed fluorapatite formation in BioMin F and, to a lesser extent, the Profluorid varnish. No apatite formation was observed in the other two varnishes. MI varnish exhibited calcium fluoride formation before and after immersion, as evidenced by XRD and ^19^F MAS-NMR analysis. **Conclusions**: The novel BioMin F varnish potentially promotes remineralisation by providing a sustained and slow release of therapeutic ions that are essential for the formation of fluorapatite.

## 1. Introduction

Dental caries remains a significant global health concern, prompting continuous research into effective preventive strategies. Fluoride-based products have emerged as a cornerstone in caries prevention, with fluoride toothpastes, rinses, and professionally applied varnishes demonstrating significant efficacy in reducing tooth decay [1,2,3]. There have been several studies that exemplify the comparable efficacy of professional varnish application to fissure sealant placement as a preventative mechanism [4]. The mechanism of action primarily involves the conversion of hydroxycarbonated apatite to fluorapatite, which exhibits greater resistance to acid dissolution [5,6].

Preventive care is essential for promoting optimal oral health, as it is cost-effective and capable of reducing the need for inpatient dental treatment [6,7]. The Delivering Better Oral Health toolkit and Jimmy Steele report acknowledge the importance of prevention over operative intervention [8]. Caries research has established the importance of early remineralisation strategies for the management of the incipient carious lesions including white spot lesions (WSLs) and non-cavitated enamel surface lesions [9,10] within a minimally invasive management philosophy.

While the effectiveness of fluoride is well-established, recent research has focused on optimising its delivery and enhancing its remineralisation potential [11]. The combination of fluoride and calcium with phosphate sources is a promising approach. It promotes more effective remineralisation, particularly in high-risk individuals with limited salivary calcium and phosphate [12,13].

Several calcium and phosphate delivery systems have been developed and incorporated into fluoride varnishes, including bioactive glasses (e.g., NovaMin), amorphous calcium phosphate stabilised by casein phosphopeptide, hydroxyapatite, and functionalised tricalcium phosphate (fTCP). These systems aim to provide a synergistic effect with fluoride, enhancing remineralisation and clinical efficacy [14,15,16].

The dental professional has excellent confidence based on studies which demonstrate optimal remineralisation of white spot lesions (WSLs). Studies confirm the synergistic application of casein phosphopeptide–amorphous calcium phosphate (CPP-ACP) in conjunction with fluoride [17]. Bioactive glass (BAG)-containing products have proven efficacy as an alternative for promoting the remineralisation of WSLs [18,19].

Bioactive glasses undergo slow and sustained dissolution in the presence of oral fluids to release calcium and phosphate ions, which encourage long-term remineralisation [19]. The released ions create a favourable alkaline environment for the formation of apatite and prevent further demineralisation. This steady dissolution outperforms the early calcium burst observed in other calcium-based materials [20]. This elevated pH level is beneficial for enhancing mineral deposition, while simultaneously inhibiting the dissolution of existing tooth enamel [20,21].

Fluoride-containing BAGs, such as BioMin F, support the formation of fluorapatite (FAp) crystals, which are more chemically stable than hydroxyapatite (HAp) in acidic environments. This enhanced stability provides greater resistance to acid-induced dissolution, as FAp has a lower critical pH of 4.5 compared to HAp’s critical pH of 5.5, at which dissolution occurs in the oral cavity [19,20].

Mohammed et al. (2013) [22] reported that fluoride-substituted apatite is formed when fluoride concentrations in solution are 45 ppm or lower, as confirmed by ^19^F MAS-NMR analysis. The team conclusively demonstrated that at fluoride concentrations exceeding 45 ppm, i.e., elevated fluoride levels, an increasing amount of calcium fluoride CaF_2_ is precipitated instead of the preferred fluoride-substituted apatite [22].

Similarly, Ren et al. (2019) [23] investigated the fluoride sorption mechanisms of nanosized hydroxyapatite, a material recognised for its efficiency in fluoride removal from contaminated water. Their study confirms the findings of Mohammed et al. that CaF_2_ precipitates form at high fluoride concentrations and also identifies a correlation between lower fluoride levels and the generation of FAp-like precipitates [23].

The availability of free fluoride and calcium ions necessary for FAp formation is diminished with CaF_2_ formation. Hence, the development of a fluoridated-apatite phase is advantageous as it helps retain the apatitic structure and lowers enamel solubility [22].

This delicate balance underscores the importance of optimising the composition of fluoride varnishes to maximise their cariostatic potential. This study aims to investigate the bioactive properties of four commercially available fluoride varnishes, three of which contain additional calcium and phosphate sources. The ion release profiles and potential for apatite formation of these varnishes will be analysed to elucidate their relative efficacy in promoting remineralisation and preventing demineralisation. The findings of this study may contribute to the development of more effective preventive dental materials and inform clinical recommendations for caries prevention strategies.

## 2. Materials and Methods

### 2.1. Dental Varnish Groups

In this study, four dental varnishes were investigated. including a newly developed varnish containing BioMin F, a low-fluorine BAG commonly used in toothpaste formulations that is designed to slowly release calcium, phosphate, and fluoride ions. The glass itself contains 10,600 ppm of fluoride [24,25]. Like all bioactive glasses, BioMin F raises the pH because of ion exchange of H^+^ for Ca^2+^ and Na^+^ ions [21]. All varnishes in this study contain an additional source of sodium fluoride (5% NaF by weight). The three commercially available varnishes include additional sources of calcium and phosphate: one has tricalcium phosphate, one has casein phosphopeptide–amorphous calcium phosphate (CPP-ACP), and the final one is a standard NaF varnish [26]. These varnishes were chosen because they each contain 5% NaF and have the potential to utilise calcium and phosphate in reactions, which are relevant to the study’s objectives.

### 2.2. Extraction of Active Ingredients from the Varnish

To prepare powdered varnish for analysis, five 0.5 mL sachets from each varnish group were placed in 15 mL centrifuge tubes. Each tube was manually agitated with 10 mL of ethanol and centrifuged at 3000 rpm for 4 min. The MI varnish required additional centrifugation to settle the solids. The supernatant was removed and the sediment solids were resuspended and agitated in 3 mL of ethanol. The resulting solutions were transferred to 50 mL glass beakers for overnight evaporation at room temperature. The resulting desiccated varnish white powders were collected in 0.5 mL Eppendorf tubes. These powders were analysed using X-Ray diffraction (XRD), attenuated total reflection Fourier-transform infrared spectroscopy (ATR-FTIR), and magic-angle spinning nuclear magnetic resonance (MAS NMR) to characterise the active varnish components before immersion. This extraction process followed a similar approach of isolation and exposure to various media (Tris Buffer, AS4, and AS7) to a previous study on varnishes [26].

### 2.3. Preparation of Immersion Media

Tris Buffer: A total of 15.090 g of tris-(hydroxymethyl) aminomethane (Sigma-Aldrich, St. Louis, MO, USA) was dissolved in 800 mL of deionised water, 44.2 mL of 1 M hydrochloric acid (HCl) was added, and it was heated at 37 °C for overnight incubation. The pH was adjusted to 7.30 with 1 M HCl, and the final volume was 2.0 L. Precise measurements were taken using the Oakton pH meter (Seven2Go Pro pH meter, Mettler-Toledo Ltd., Leicester, UK), and meticulous preparation and storage ensured consistent solutions for accuracy in experiments.

Artificial Saliva Solutions: Artificial saliva solutions, both demineralising and remineralising buffers, were prepared following the method outlined by Ten Cate et al. [27,28]. Both solutions were prepared to a final volume of 2.0 L, with approximately 0.65 g of sodium aside added to inhibit bacterial growth. All chemicals below were sourced from Sigma-Aldrich (St. Louis, MO, USA).

- Demineralising Buffer (AS4): A total of 0.4411 g of calcium chloride dihydrate (CaCl_2_⋅2H_2_O) and 0.245 g of potassium dihydrogen phosphate (KH_2_PO_4_) were dissolved in 800 mL of deionised water. Then, 5.72 mL of acetic acid was added, and the pH was adjusted to 4.0 using 0.5 M potassium hydroxide (KOH).
- Remineralising Buffer (AS7): A total of 0.4411 g of CaCl_2_⋅2H_2_O, 0.245 g of KH_2_PO_4_, 9.532 g of HEPES, and 19.386 g of potassium chloride (KCl) were dissolved in 800 mL of deionised water. The pH was adjusted to 7.0 using 0.5 M KOH.

### 2.4. Sample Preparation

A 22 × 22 mm borosilicate glass coverslip and thickness No. 1 (VWR international, Radnor, PA, USA) was weighed before and after application of a thin coat of varnish, ensuring a uniform deposition of 0.0370 ± 0.0008 g measured using a precision analytical balance (Mettler Toledo HK 160, Mettler-Toledo Ltd., Leicester, UK). Eight samples were prepared per varnish group, of which six were immersed in solution. Two samples were immersed in 10 mL of Tris buffer, two in AS4 medium, and two in AS7 medium, while two were retained as non-immersed controls (0 h). The duplicates for each medium were immersed for durations of 2, 4, 6, and 24 h, with the media refreshed at each time point. This process was repeated for all varnish groups and media types.

### 2.5. Post-Immersion Media Analysis

The post-immersion solutions were analysed for changes in pH, fluoride, calcium, and phosphorus release at 2, 4, 6, and 24 h. Fluoride concentrations were measured using an ion-selective electrode (ELIT 221, Nico2000 Ltd., London, UK), calibrated with 0, 10, 100, and 1000 ppm fluoride solutions. The calibration curve (E vs. log [F]) showed a linear relationship (R^2^ ≈ 0.999). The pH changes were measured using a calibrated Seven2Go S8-Kit pH meter (Mettler Toledo Ltd., Leicester, UK) with an InLab Expert Go-ISM electrode. Calcium, phosphorus, and sodium release were quantified using Inductively Coupled Plasma–Optical Emission Spectroscopy (ICP-OES, Thermo Fisher iCAP 7400 Duo, Thermo Fisher Scientific, Waltham, MA, USA) at wavelengths of 422.673 nm for calcium and 213.618 nm for phosphorus, with a calibration range of 0–80 ppm. Prior to ICP-OES analysis, 1 ml of 1 mol nitric acid was added to each sample to dissolve any particulate matter.

### 2.6. Surface Analysis of Coverslips

#### 2.6.1. X-Ray Diffraction (XRD)

XRD was performed on the pre- and post-immersion varnish using a PANalytical CubiX^3^ X-Ray diffractometer (Malvern PANalytical, Worcestershire, UK), operated at 45 kV and 40 mA with Cu Kα radiation and a Ni filter. Diffraction data were collected over a 2θ range of 5–70° in continuous scanning mode, with samples spinning during acquisition. The analysis aimed to detect a reduction in crystalline sodium fluoride (NaF) and the potential formation of crystalline FAp or CaF_2_. Post-immersion samples were dried for 72 h prior to analysis, and their diffraction patterns were compared to a hydroxyapatite reference (Sigma-Aldrich, St. Louis, MO, USA) to identify crystalline changes. This approach allowed for the assessment of structural modifications in response to immersion at different time points.

#### 2.6.2. Attenuated Total Reflectance–Fourier Transform Infrared Spectroscopy (ATR-FTIR)

Analysis was conducted using ATR-FTIR (Frontier FTIR, Perkin Elmer, Waltham, MA, USA) on extracted varnish powders pre-immersion and on dried varnish coverslips post-immersion in Tris buffer, AS4, and AS7. Pure hydroxyapatite powder served as a reference. This technique was used to identify functional groups by assessing molecular vibrations in pre-immersion powders and post-immersion coverslips. However, the spectra were predominantly influenced by the rosin resin, which may have masked other chemical changes.

#### 2.6.3. Magic Angle Spinning Nuclear Magnetic Resonance (MAS-NMR)

MAS-NMR analysis was performed on varnish samples before and after immersion to investigate the chemical speciation of phosphorus and fluorine. A 600 MHz Bruker spectrometer (Bruker, Billerica, MA, USA) operating at 14.1 Tesla was used for solid-state MAS-NMR experiments. ^19^F and ^31^P MAS-NMR spectra were acquired at resonance frequencies of 564.7 MHz and 242.9 MHz, respectively. Samples were packed in 2.5 mm zirconia rotors and spun at 22 kHz (^19^F) and 12 kHz (^31^P) using a standard double-resonance Bruker probe with fluorine-free background. Chemical shift references were established using 1 M aqueous NaF solution −120 ppm for ^19^F and 85% H_3_PO_4_ solution for ^31^P. Experiments were conducted with a 60 s recycle delay, and species content was quantified by integrating the area under the NMR peaks. Notably, ^19^F MAS-NMR allowed differentiation between fluorapatite and hydroxyapatite, whereas X-Ray diffraction alone is unable to make the distinction. This comprehensive MAS-NMR analysis provided detailed information on the chemical attributes and speciation of the varnish samples before and after immersion.

## 3. Results

The results are presented in two main sections. First, we describe the characterisation of the four varnishes before immersion, including XRD and NMR analysis to identify crystalline phases and chemical composition. Second, post-immersion results are presented, including XRD and NMR analysis of varnish-coated coverslips, alongside ion release profiles measured by F-ISE and ICP-OES.

### 3.1. Before Immersion Studies–Varnish Characterisation

**Figure 1:**
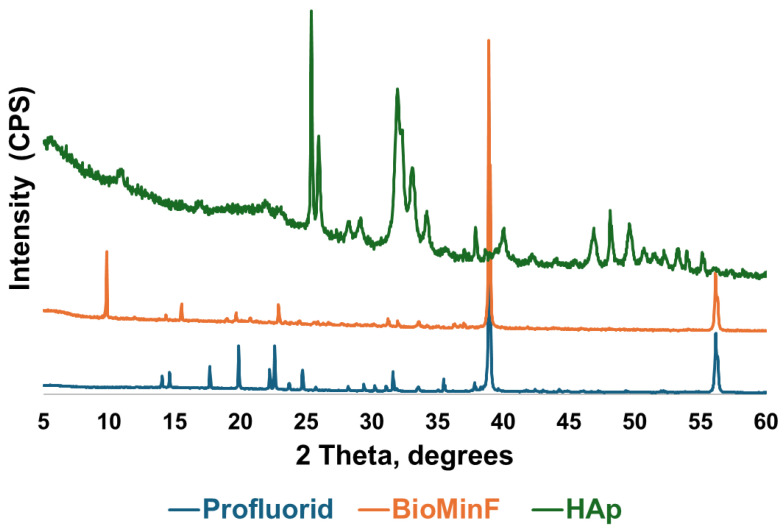
The XRD patterns of extracted BioMin F, Profluorid varnishes, alongside a hydroxyapatite reference.

Crystalline peaks at approximately 38.8° and 56.5° 2θ were observed in both varnishes (Figure 1). BioMin F additionally displayed a peak at approximately 9.8° 2θ. No crystalline peaks corresponding to those observed in the HAp reference standard (25.3°, 25.9°, 31.9°, 33.1°, and 34.1° 2θ) were identified in either varnish prior to immersion.

**Figure 2:**
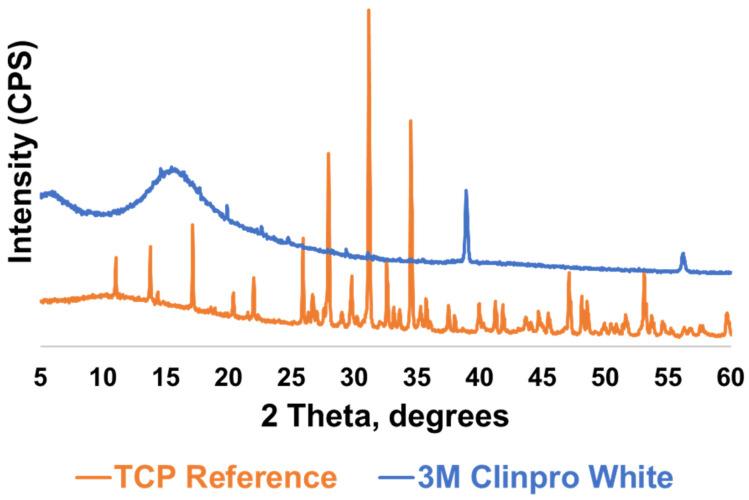
The extracted Clinpro White varnish compared to a tricalcium phosphate reference pattern.

An amorphous peak was detected at around 15.8° 2θ, and crystalline peaks at 38.7° and 55.8° 2θ were identified in the varnish (Figure 2). The β-TCP reference displayed characteristic crystalline peaks at approximately 28°, 31°, and 34° 2θ. No peaks corresponding to the β-TCP reference pattern were identified in the extracted Clinpro White varnish.

**Figure 3:**
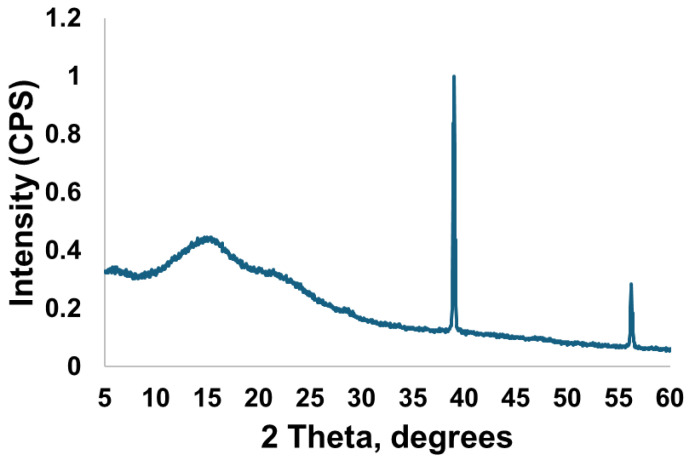
The XRD pattern of the extracted MI varnish.

A broad amorphous hump centred at approximately 15.3° 2θ was observed, alongside a faint broad feature at approximately 28.6° 2θ with crystalline peaks at 38.8^o^ and 56.2^o^ 2θ (Figure 3).

### 3.2. ^19^F and ^31^P MAS-NMR Analysis Prior to Immersion

**Figure 4:**
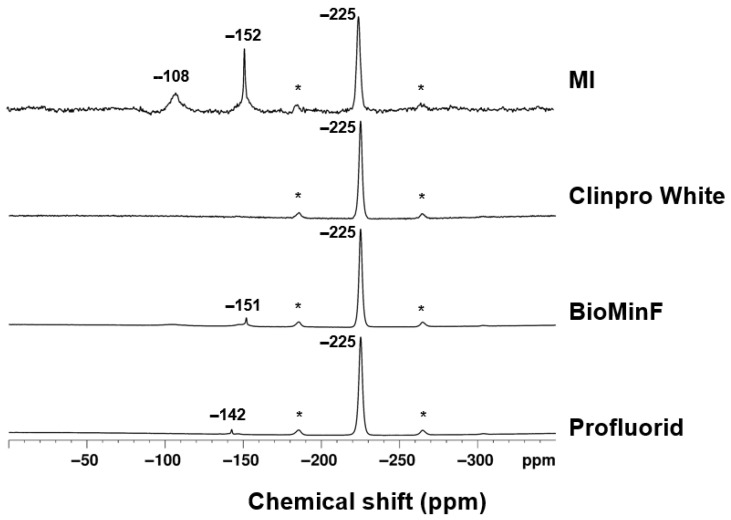
The ^19^F MAS-NMR spectra of the varnishes prior to immersion. Spinning sidebands are marked by an asterisk (*).

The ^19^F MAS-NMR spectra for all four varnishes exhibit a peak at −225 ppm, indicative of crystalline NaF (Figure 4). The ^19^F NMR spectra showed additional signals at −151 ppm for BioMin F varnish, −152 ppm for MI varnish, and −142 ppm for Profluorid varnish. Additionally, the MI varnish spectrum showed a peak at −108 ppm.

**Figure 5:**
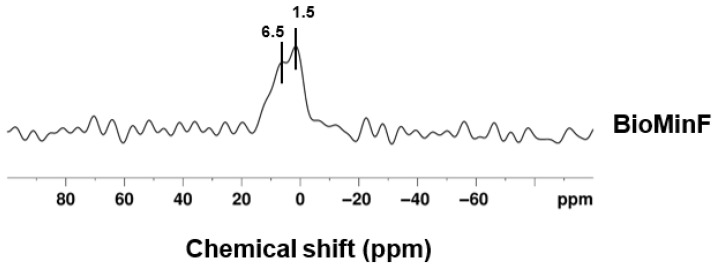
The ^31^P MAS-NMR of BioMin F varnish prior to immersion.

The ^31^P MAS-NMR spectrum displays two distinct peaks rather than the single broad peak typically associated with sodium-containing fluoride bioactive glasses like BioMin F (Figure 5).

### 3.3. Post-Immersion Studies

#### X-Ray Diffraction

**Figure 6:**
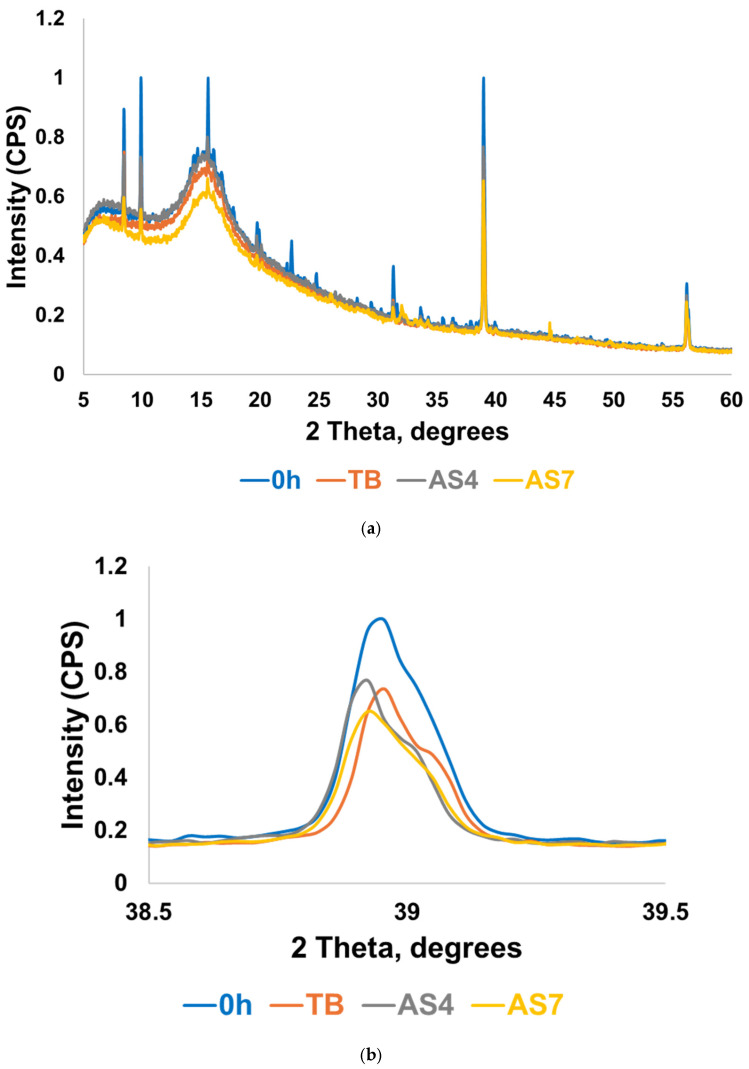
(**a**) The XRD pattern of the BioMin F varnish before and after immersion in all media. (**b**) highlights the primary sodium fluoride peak at 38.7° 2θ in greater detail.

The BioMin F varnish displayed diffraction peaks at 38.7° 2θ, 56.0° 2θ and a minor peak at 33.5° 2θ (Figure 6). Similar peaks were also identified in the Profluorid and Clinpro White varnishes.

**Figure 7:**
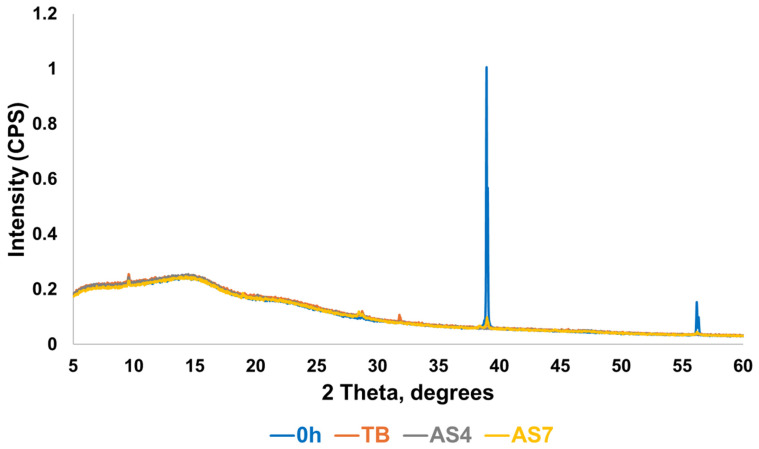
The XRD pattern of the MI varnish before and after immersion in TB AS4 and AS7.

XRD analysis of MI varnish revealed strong diffraction peaks at 38.7° and 56° 2θ in the non-immersed sample (0 h), which were markedly reduced or absent following immersion in TB, AS4 and AS7 media (Figure 7).

### 3.4. ^19^F and ^31^P MAS-NMR Analysis Post-Immersion 

**Figure 8:**
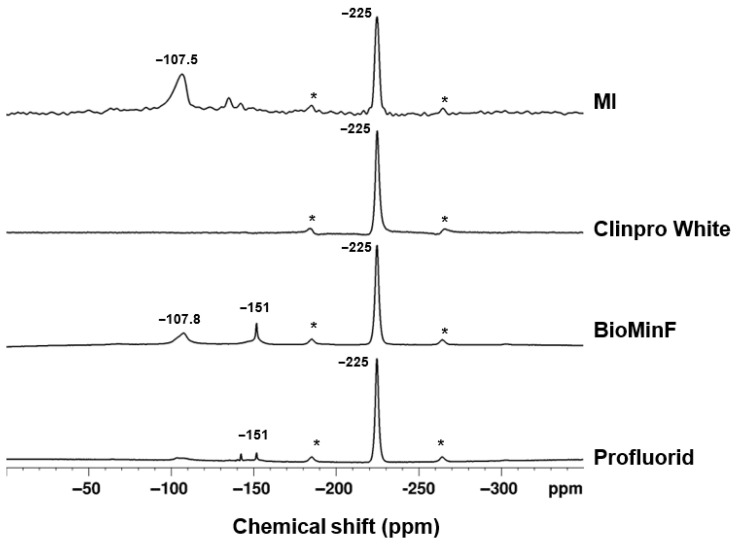
The ^19^F MAS-NMR spectra of the varnishes post-immersion. Spinning sidebands are marked by an asterisk (*).

The ^19^F MAS-NMR spectrum of BioMin F varnish (Figure 8) exhibited two distinct peaks at approximately −151.7 and −107.8 ppm.

**Figure 9:**
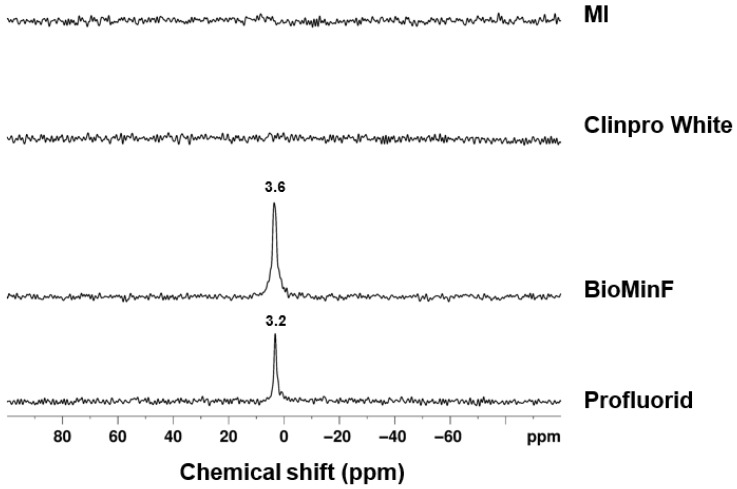
The ^31^P MAS-NMR spectra of all the varnishes post-immersion.

The BioMin F varnish exhibited a chemical shift at 3.6 ppm (Figure 9).

Profluorid varnish revealed a peak at approximately 3.2 ppm, which was sharper than the broad peak observed for the BioMin F varnish. However, the spectra for the GC MI and Clinpro White varnish did not show any detectable ^31^P signal.

### 3.5. Post-Immersion Studies of the Media

#### pH Analysis of Post-Immersion Media

The pH analysis aimed to assess whether BioMin F glass in the varnish formulation would elevate pH compared to other varnishes. Results showed a slight, insignificant pH increase after 24 h in AS4 media (pH 4.0 to 4.1), indicating a low BioMin F glass content. This data has not been included in the paper.

### 3.6. Fluoride Release in TB, AS4 and AS7

**Figure 10:**
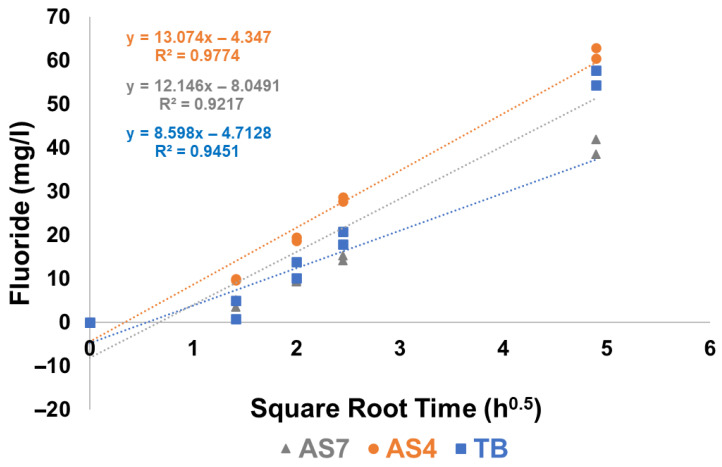
Cumulative fluoride release from MI varnish in TB, AS4 and AS7 as a function of square root time.

**Figure 11:**
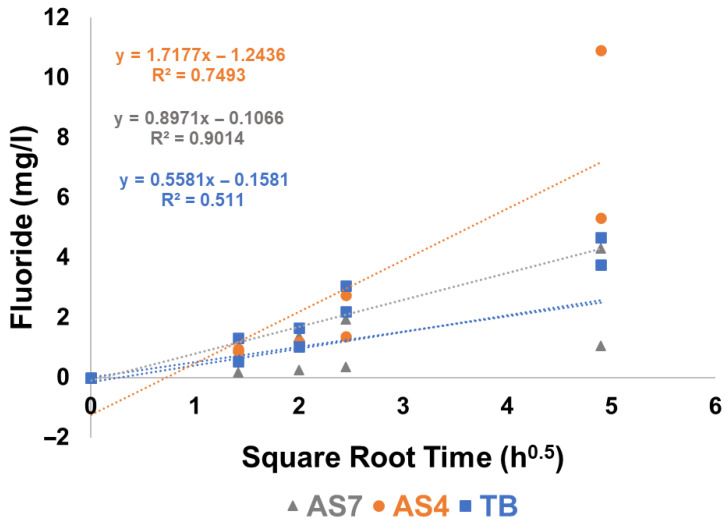
Cumulative fluoride release from Clinpro White varnish in TB, AS4 and AS7 as a function of square root time.

In all cases, the measured fluoride concentration was linear when plotted against the square root of time for all four varnishes in all three immersion media (Figure 10 and Figure 11). This linear relationship is consistent with a diffusion-controlled release mechanism governed by Fick’s Second Law of Diffusion, whereby cumulative release is proportional to the square root of time—a pattern characteristic of matrix-type diffusion systems [29].

**Figure 12:**
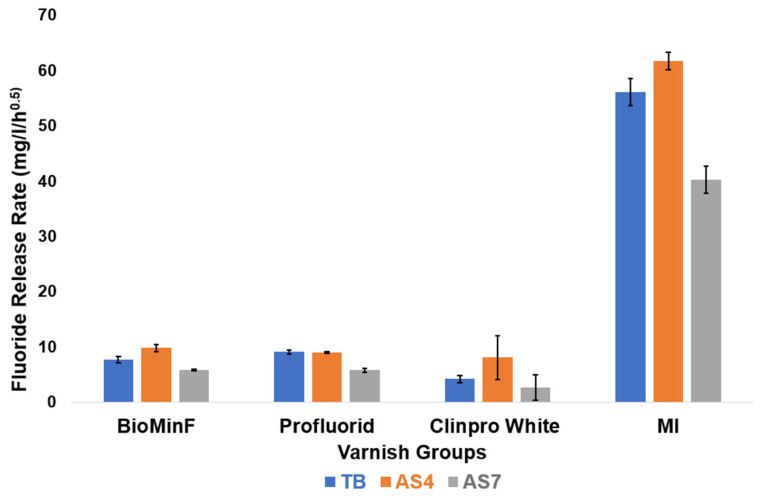
Fluoride release rate for BioMin F, Profluorid, Clinpro White and MI varnishes in TB, AS4 and AS7.

MI varnish consistently exhibited higher fluoride release across all three immersion media compared to the other varnishes (Figure 12). Notably, all four varnishes demonstrated lower measured free fluoride concentrations in AS7 relative to TB or AS4. Profluorid Varnish and BioMin F displayed comparable fluoride release rates in TB, AS4, and AS7, suggesting similar release kinetics despite their different formulations.

### 3.7. Sodium, Calcium and Phosphate Release (ICP-OES)

**Figure 13:**
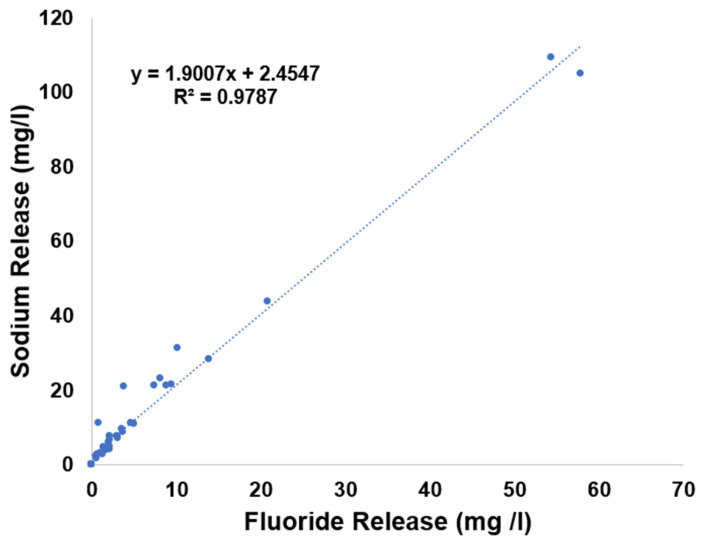
Correlation between fluoride and sodium release across all varnish groups in TB, derived from ISE and ICP-OES measurements respectively.

A positive linear relationship was observed between fluoride and sodium release across all varnish groups in TB media (R^2^ = 0.9787, Figure 13).

**Figure 14:**
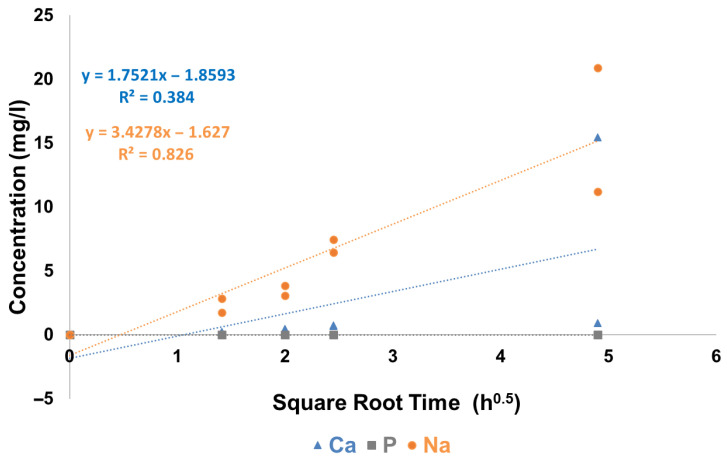
Ca, P and Na release from Clinpro White varnish in TB as a function of square root time.

No phosphorus release was detected in TB after immersion of Clinpro White varnish at any time point, though very low amounts of calcium were detected (Figure 14). Sodium release increased progressively over time, while calcium and phosphorus releases remained negligible throughout the immersion period.

## 4. Discussion

This study investigated how the formulation and ion release kinetics of fluoride varnishes, supplemented with calcium and phosphate, influence enamel remineralisation. Our findings highlight that product efficacy depends not only on fluoride concentration, but critically on the interplay of active and inactive components. Previous studies have emphasised the importance of high fluoride concentration in dental varnishes for therapeutic benefit. However, this study investigated the effectiveness of different formulations of fluoride varnishes supplemented with calcium and phosphate, specifically underscoring the critical importance of ion release kinetics in promoting enamel remineralisation for potential preventive dental care applications.

Figure 1 shows the XRD patterns of the extracted VOCO varnishes (BioMin F and Profluorid) compared against a hydroxyapatite reference to assess whether any reaction had occurred within the packaging. The diffraction patterns for the varnishes showed prominent peaks at 38.7° and 56.0° 2θ, confirming the presence of crystalline NaF in the varnishes as expected [26]. The absence of hydroxyapatite peaks indicated that the active ingredients in the varnishes did not react, demonstrating the stability of the formulations. The peak observed at 9.8° 2θ in BioMin F is consistent with the (020) reflection of brushite, though shifted to a lower angle compared to the characteristic reflection reported at 11.7° 2θ for pure brushite [30,31]. This shift may suggest a modified or impure brushite phase, potentially arising from the presence of other components within the varnish formulation.

Figure 2 shows a broad amorphous hump at approximately 15.8° 2θ, which may be attributed to the rosin-based carrier matrix of the varnish, which is inherently non-crystalline. A β-TCP reference was included for comparison, given that the active calcium phosphate ingredient in Clinpro White is fTCP, which is derived from β-TCP through surface modification with fumaric acid [16]. The absence of peaks corresponding to the β-TCP reference in the Clinpro White varnish suggests that crystalline fTCP was not detectable.

Figure 3 shows the XRD pattern of the extracted MI varnish, which displayed a faint broad peak around 28° 2θ, characteristic of the CaF_2_ crystal phase [11]. This implies that a portion of the varnish may have reacted within the sachet, leading to the formation of CaF_2_. This presents as a slight disadvantage since some of the free fluoride and calcium ions have been utilised in the creation of CaF_2_, subsequently diminishing the concentration of ions available for the formation of fluorapatite following application in the mouth.

Figure 4 shows the spectra of the extracted active ingredients prior to immersion using ^19^F MAS-NMR, which revealed consistent findings, with all samples showing a chemical shift of −225 ppm for sodium fluoride, as anticipated. There were additional peaks seen at −151 ppm, −152 ppm, and −142 ppm, which are possibly attributed to fluorine complexes with the resin. The MI varnish also showed a chemical shift at −108 ppm, which is characteristic of CaF_2_ [23], aligning with the XRD data in Figure 2.

The BioMin F glass exhibited a broad ^31^P peak at 7.0 ppm, indicative of orthophosphate charge-balanced equally by calcium and sodium. However, in Figure 5, the ^31^P MAS-NMR spectrum of the BioMin F varnish displays two distinct peaks: a broad peak at 6.5 ppm and a sharper peak at 1.5 ppm. The latter is attributed to brushite, which typically exhibits a chemical shift around 1.3 ppm [32].

This suggests that brushite, a compound regarded as a precursor to apatite formation, was produced during varnish formulation, which likely took place under acidic conditions [33]. Notably, bioactive glasses generally form apatite in basic media, with fluorine-containing bioactive glasses like BioMin F typically producing fluorapatite [34].

In the post-immersion study, the coverslips were analysed by XRD and MAS-NMR to determine whether apatite or calcium fluoride formation had occurred. In XRD analysis for the coverslips coated with BioMin F varnish (Figure 6), strong diffraction peaks at 38.7° 2θ, 56.0° 2θ, and minor peaks at 33.5° 2θ were observed, all of which correspond to crystalline sodium fluoride [26]. In Figure 6b, the principal peak for sodium fluoride at 38.7° 2θ is magnified so the degree of dissolution of the sodium fluoride can be discerned more accurately. The peak observed in the non-immersed state can serve as a reference point to gauge the extent of sodium fluoride dissolution in the immersion media. Particularly, the AS7 medium demonstrates the highest degree of sodium fluoride dissolution, indicating that maximum fluoride had been taken up from the varnish in AS7 media.

In Figure 8, for the BioMin F varnish, the peak around −107.8 ppm was of interest, as it closely resembles the −108 ppm peak commonly linked to calcium fluoride [23]. The observed peak displays an asymmetric shape with a slight rightward skew, and the chemical shift for FAp is −103 ppm, which suggests the potential presence of both CaF_2_ and an apatite-like phase [23]. This aligns with the understanding that fluoride’s ability to promote net remineralisation is reliant on the availability of calcium and phosphate ions. To confirm the presence of apatite, ^31^P MAS-NMR was performed (Figure 9) and shows the disappearance of signals corresponding to brushite and the amorphous glass phase (which was seen in Figure 5), with a new peak appearing for an apatite-like phase at 3.5 ppm [35]. This suggests that the varnish underwent a reaction in AS7, leading to the development of a more stable mineral phase, enhancing its potential for enamel remineralisation.

The Voco Profluorid varnish shows a sharper chemical shift at 3.2 ppm in Figure 9, indicating the formation of a more well-ordered and crystalline apatite-like structure [35]. This finding is consistent with the broader literature on fluoride varnishes, which underscores their effectiveness in promoting remineralisation processes, as seen in the systematic review by Marinho et al. (2009) on fluoride varnishes for preventing dental caries in children and adolescents [36].

The post-immersion fluoride analysis using an ion-selective electrode demonstrated high reproducibility (Figure 10) with duplicate values agreeing within 5% for all varnishes except for the Clinpro White varnish (Figure 11). This variability is likely due to bubbling observed following the coating of Clinpro White varnish on the coverslips during sample preparation, plausibly caused by interactions between its thickening agent and ethyl alcohol content in (Table 1). Similar bubbling was imaged and documented by Cochrane et al. [37]. Evaporation of ethyl alcohol from the varnish may be hindered by the thickener, resulting in detachment and fragmentation during immersion, which disrupts fluoride ion release. Notably, Clinpro White varnish did not precipitate either CaF_2_ or fluorapatite post-immersion, as shown in Figure 8, likely because of this formulation instability. Furthermore, despite manufacturer claims of fTCP, no detectable ^31^P signal was observed in either post-immersion analysis (Figure 9), aligning with pre-immersion XRD data (Figure 2) showing no detectable TCP.

BioMin F, Profluorid, and Clinpro White varnishes demonstrated comparatively lower cumulative fluoride release across all media after 24 h, in contrast to the MI varnish, which exhibited much higher fluoride release across all media and timepoints (Figure 12). This is consistent with the XRD data (Figure 7), where a marked reduction in the sodium fluoride diffraction line was observed following immersion across all tested media. These findings are in agreement with Cochrane et al. (2014), who reported that MI varnish had the highest fluoride release in comparison to other varnish groups [37]. Notably, in Figure 8, the MI varnish shows a prominent chemical shift at −107.5 ppm, which closely aligns with the characteristic peak position for CaF_2_ at −108 ppm [23].

This indicates that all the fluoride that has been released in the media has been taken up to form CaF_2_. Previous studies report that ACP-CPP in MI Plus remineralising paste (900 ppm F^−^) promotes FAp formation. However, in our study, the varnish was associated with CaF_2_ formation. This CaF_2_ formation is explained by the much higher fluoride concentration (22,600 mg/L) in the varnish compared to MI Plus paste. This rapid release of fluoride is what drives towards calcium fluoride formation rather than FAp [38].

The sodium and fluoride ion release were plotted against each other for all varnishes (Figure 13) to assess the correlation between their release. The observed slope (1.9) exceeded the expected stoichiometric ratio for NaF (1.21, based on 23/19 weight basis), indicating that additional sodium from the bioactive glass in BioMin F varnish was released. Variability in Clinpro White varnish ion release also contributed to this discrepancy.

These findings suggest that the inactive components (rosin/resin) play a crucial role in determining the ion release of the varnishes [39]. The results demonstrate that sustained fluoride delivery, as seen in BioMin F and Profluorid varnishes, supports apatite-like formation and enhances remineralisation potential. In contrast, the burst release pattern of MI varnish, whilst delivering high fluoride concentrations, results in CaF_2_ formation. This reinforces the importance of sustained fluoride delivery for maximum therapeutic effect, a point also highlighted by a study conducted by Shen and Autio-Gold, where they discovered that extended adherence of the varnish to the tooth’s surface, coupled with a gradual and extended fluoride release, offers long-term benefits in preventing tooth decay [39].

This also supports the Ten Cate principle that the primary effect of fluoride on caries control requires maintaining a low, constant local concentration in the oral environment to reverse demineralisation. The reliance on high fluoride content alone may not yield the desired therapeutic effect [40,41].

This study suggests that BioMin F varnish promotes remineralisation due to the presence of an acidic phosphate phase in the glass, which acts as a precursor to apatite formation and transforms into a more stable apatite-like mineral post-immersion. In contrast, GC MI varnish exhibited rapid “burst release” of fluoride, leading to the formation of CaF_2_ precipitates. The Clinpro White varnish lacked detectable tricalcium phosphate, contrary to manufacturer claims. The BioMin F, Profluorid, and Clinpro White varnishes showed a relative reduction in NaF peak intensity following immersion, though a substantial peak remained detectable even after 24 h, suggesting that a considerable proportion of NaF was retained. In contrast, GC MI varnish showed a markedly greater relative reduction in NaF peak intensity across all media.

This study was conducted using a relatively low concentration of bioactive glass within the BioMin F varnish formulation. While this allowed the influence of glass-derived ions on release behaviour and mineral formation to be observed, it may be of further scientific and clinical interest to investigate a wider range of bioactive glass loadings. Systematically varying the bioactive glass content could provide deeper insight into its role in controlling ion release kinetics and mineral phase formation, and its potential impact on enamel and dentine substrates under more clinically relevant conditions. One limitation of this study is the proprietary nature of the BioMin F glass formulation, which restricts full compositional disclosure and may impact reproducibility. While fluoride-containing bioactive glasses in the SiO_2_–P_2_O_5_–CaO–Na_2_O system are well documented, BioMin F’s patented composition prevents direct comparison with published formulations [21,42].

The established history of BioMin F in the literature supports its credibility and applying it in a dental varnish marks an innovative advance. The article addresses commonly encountered challenges such as moisture sensitivity by characterising it within the varnish resin for ion release, pH changes, and apatite formation. Despite potential challenges in replication, the research demonstrates the efficacy and appropriateness of clinical consideration of bioactive glass-containing dental varnishes. These findings reinforce the importance of considering complete formulation and sustained ion release, rather than fluoride concentration alone, when evaluating fluoride varnishes for preventive dental applications.

## 5. Conclusions

This study provides a comparative evaluation of four fluoride varnishes, demonstrating that product performance is governed not only by fluoride concentration but critically by overall formulation and ion release kinetics. Although all varnishes contained 5% NaF as the primary active ingredient, substantial differences in fluoride release behaviour were observed, highlighting the importance of resin matrices and the availability of calcium and phosphate ions. Varnishes associated with more sustained fluoride release showed evidence of apatite-like mineral formation, whereas rapid fluoride release was associated with calcium fluoride formation. From a clinical perspective, these findings indicate that reliance on fluoride concentration alone may be insufficient when selecting preventive varnishes. Instead, varnish formulation and the capacity to deliver fluoride in a sustained manner alongside bioavailable calcium and phosphate should be considered, as these factors are more closely aligned with apatite-like mineral formation and the intended remineralisation benefit.

## 6. Patent

One of the applicants, RH, is an inventor on the patent owned by a former institution, Imperial College Innovations, “Multicomponent Glasses for Use in Personal Care Products” WO 2011/000866A2 and this patent is licenced to BioMin Technologies Ltd. (London, UK).

## Figures and Tables

**Table 1:** List of active and inactive components of the fluoride varnishes used in this study.

Varnishes	Active Ingredients	Inactive Ingredients
Profluorid Varnish (Voco GmbH, Cuxhaven, Germany)	NaF	ethanol, colophony, xylitol, artificial flavours
Profluorid varnish + BioMin F, (Voco GmbH, Cuxhaven, Germany)	NaF + BioMin F glass	Identical to the Profluorid varnish but with BioMin F glass
MI Varnish, (GC, Tokyo, Japan)	NaF + CPP-ACP	Polyvinyl Acetate, Hydrogenated rosin, Ethanol, Silicon Dioxide
Clinpro™ White varnish, (3M ESPE, St. Paul, MN, USA)	NaF + modified TCP	Pentaerythritol glycerol ester of colophony resin, n-Hexane, Ethyl alcohol, Flavour enhancer, Thickener, Food-grade flavour

## Data Availability

The original contributions presented in the study are included in the article. Further inquiries can be directed to the corresponding authors.
